# The Median Palatine Suture: A Comprehensive Review With Clinical Considerations

**DOI:** 10.7759/cureus.94695

**Published:** 2025-10-16

**Authors:** Emma R Lesser, Shogo Kikuta, Blair M Barton, Rarinthorn Samrid, Hotaka Kawai, R. Shane Tubbs, Joe Iwanaga

**Affiliations:** 1 Department of Neurosurgery, Clinical Neuroscience Research Center, Tulane University School of Medicine, New Orleans, USA; 2 Department of Surgery, Dental and Oral Medical Center, Kurume University School of Medicine, Kurume, JPN; 3 Department of Otorhinolaryngology, Ochsner Health System, New Orleans, USA; 4 Department of Anatomy, Khon Kaen University, Khon Kaen, THA; 5 Department of Oral Pathology and Medicine, Okayama University, Okayama, JPN; 6 Department of Neurology, Clinical Neuroscience Research Center, Tulane University School of Medicine, New Orleans, USA; 7 Department of Structural and Cellular Biology, Tulane University School of Medicine, New Orleans, USA; 8 Department of Neurosurgery, Ochsner Neuroscience Institute, Ochsner Health System, New Orleans, USA; 9 Department of Anatomical Sciences, St. George’s University, St. George’s, GRD; 10 Department of Surgery, Tulane University School of Medicine, New Orleans, USA; 11 Department of Neurosurgery, Dental and Oral Medical Center, Kurume University School of Medicine, Kurume, JPN; 12 Department of Anatomy, Kurume University School of Medicine, Kurume, JPN

**Keywords:** anatomy, cleft palate, comparative anatomy, cranial sutures, embryology, forensic age assessment, palate

## Abstract

The median palatine suture (MPS) is an articulation in the midsagittal plane in the oral cavity's roof that divides the palate into two halves. Its anterior and posterior segments are formed by the fusion of the maxillary palatine processes and the horizontal plates of the palatine bones, respectively. This review synthesizes the classical understanding of the MPS with recent multidisciplinary advancements to provide a contemporary and holistic perspective. We delve into its comparative anatomy and evolutionary origins, and further detail the molecular and cellular mechanisms governing its development, maturation, and response to mechanical force. The discussion extends to modern imaging modalities for assessing sutural patency and maturation, encompassing cone-beam computed tomography (CBCT), magnetic resonance imaging (MRI), and the application of artificial intelligence (AI) for automated analysis. Clinically, the MPS is significant across diverse fields; its role is discussed in the context of pathology, such as cleft palate, its utility in forensic age estimation, and its central importance in contemporary orthodontic and surgical interventions, notably microimplant-assisted rapid palatal expansion (MARPE). By consolidating the current state of knowledge and identifying key directions for future research, this review serves as an essential resource for clinicians and researchers engaged in maxillofacial surgery, orthodontics, and forensic science.

## Introduction and background

**Figure 1:**
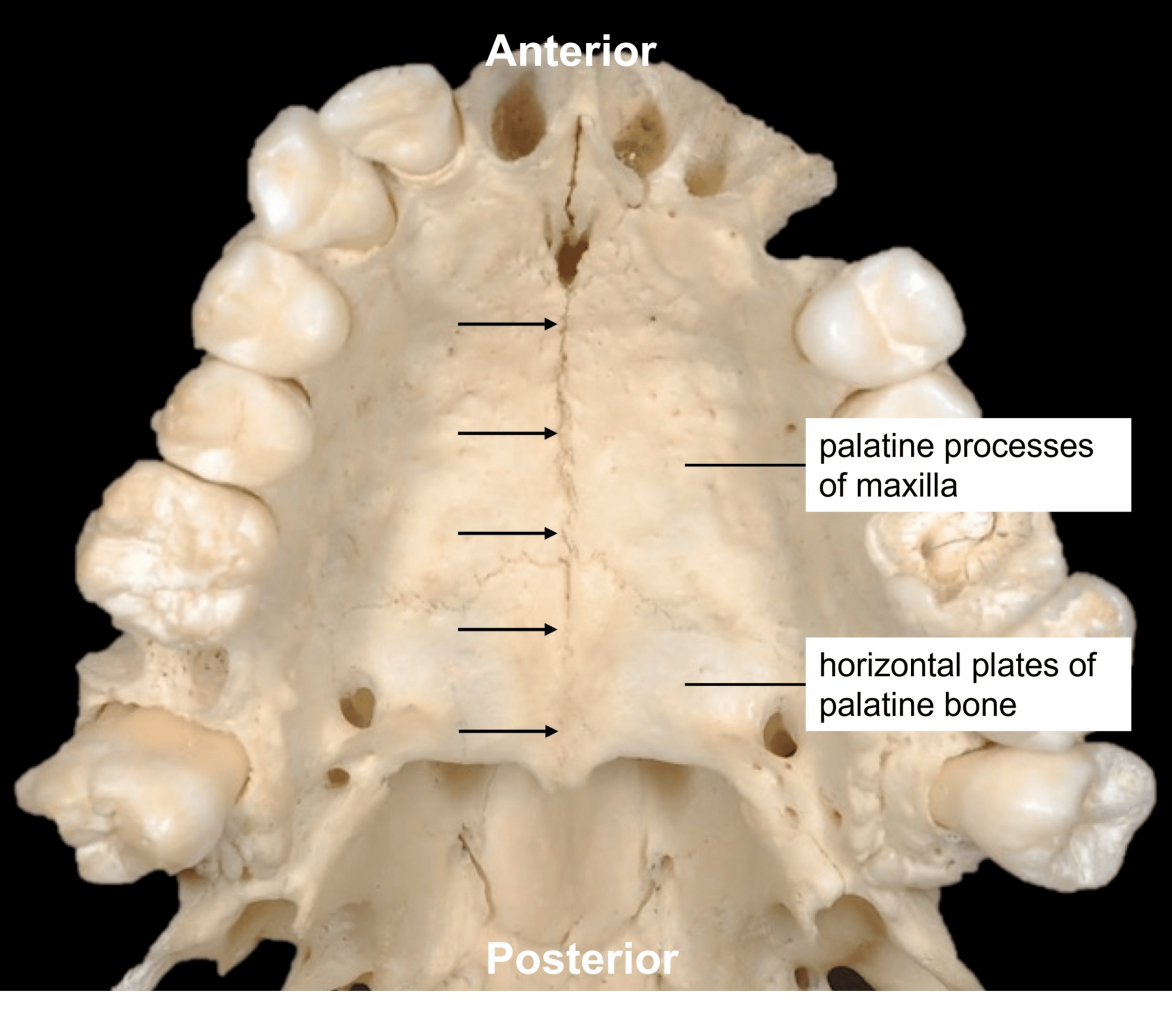
Median palatine suture (arrows). Source: Original image obtained from a cadaveric specimen in the present study.

The palate, or roof of the oral cavity, comprises two sections: the hard palate and the soft palate [1]. The pharyngeal muscles and mucosa form the soft palate that connects the oral cavity to the pharynx [2]. The bony palate, which is a name for the palatal region of the skull, is formed by the fusion of four bones - the bilateral palatine processes of the maxillae and bilateral horizontal plates of the palatine bones [3], which converge along the midsagittal plane to form the roof of the oral cavity [4]. The median articulation formed from this junction is termed the median palatine suture (MPS) (Figure 1).

The MPS is continuous with the intermaxillary suture (IMS) between the maxillary central incisors and extends anteroposteriorly from the palatine processes of the maxillae to the horizontal plates of the palatine bones via the transverse palatine suture (TPS). This horizontal bony fusion joins the palatine bones posteriorly with the maxillary palatine processes anteriorly; the TPS and MPS together form a cruciform suture [4].

Beyond its structural role in humans, the palate represents a key anatomical innovation in vertebrate evolution, with the secondary palate developing convergently in mammals and crocodylians to separate the oral and nasal cavities, thereby facilitating simultaneous breathing and feeding, a function critical for neonatal suckling, and enabling the complex oral pressure modulation required for articulated speech [5-7]. Recent advances in molecular biology have elucidated the key signaling pathways, e.g., Wingless/Integrated (Wnt) and bone morphogenetic protein (BMP), and suture stem cells that orchestrate the MPS's development, maturation, and osteogenic response to mechanical forces [8-11]. This growing biological understanding is complemented by advanced imaging techniques, such as cone-beam computed tomography (CBCT), which permit precise, individualized assessment of suture maturation [12-15]. Consequently, the MPS is a focal point in diverse fields, from forensic science, where its obliteration is used for age estimation [16-18], to clinical practice, where its manipulation through interventions like microimplant-assisted rapid palatal expansion (MARPE) has revolutionized the treatment of maxillary transverse discrepancies [19,20]. In light of these multifaceted roles and the rapid expansion of relevant knowledge, this review aims to synthesize classical anatomical descriptions of the palate with contemporary findings in molecular biology, advanced imaging, and clinical intervention, while also identifying promising directions for future research.

## Review

Search strategy and scope

To ensure a comprehensive foundation for this review, a literature search was conducted across several major electronic databases, including PubMed/MEDLINE, Scopus, and Google Scholar. The search strategy employed a combination of keywords and Medical Subject Headings (MeSH) terms, including "median palatine suture," "midpalatal suture," "palatal anatomy," "palatal expansion," "MARPE," "cleft palate," and "forensic age estimation." The literature was selected based on its relevance to the core themes of this review, encompassing embryology, molecular biology, advanced imaging, clinical interventions, and forensic applications. Both historical seminal papers and recent research articles were included to provide a balanced and contemporary perspective. The synthesis of this information was performed narratively to construct a holistic and multidisciplinary overview of the MPS.

Comparative anatomy and evolutionary context of the palate

The palate represents a key anatomical innovation in vertebrate evolution, exhibiting remarkable diversity in structure and function across major clades. In amniotes, including mammals, birds, and reptiles, the development of a secondary palate, which separates the oral and nasal cavities, is a striking example of convergent evolution [5,6]. This structure has independently arisen in mammals, crocodylians, and some lizards, facilitating simultaneous feeding and breathing and supporting ecological diversification [5]. While this convergent pattern is prominent in certain lineages, other reptiles and birds exhibit distinct palatal morphologies that reflect their own specific adaptations and functional demands [5,21].

The evolutionary trajectory of the palate is further illuminated by fossil evidence. Early crocodylomorphs exhibited open palates with anteriorly positioned choanae, while later forms developed ossified secondary palates and posteriorly shifted choanae, likely in response to dietary shifts and semi-aquatic lifestyles [5]. In mammalian ancestors (therapsids), the emergence of robust choanal folds and their anterior fusion provided the foundation for the bony secondary palate, which evolved independently multiple times [6]. The soft palate, derived from muscularized choanal folds, contributed to the formation of a sealed oral cavity, enhancing neonatal suckling and respiratory efficiency [6]. Functional and mechanical analyses underscore the evolutionary significance of the secondary palate. In mammals, the fusion of palatal shelves at the midline confers substantial increases in torsional strength and stiffness to the rostrum, supporting the development of complex masticatory mechanisms and dietary specializations [22]. Among primates, palate shape is strongly influenced by body size and diet, with evolutionary shifts in palate morphology accompanying changes in feeding strategies and vocalization patterns [23].

Recent advances in developmental biology have provided further insight into the molecular and cellular mechanisms underlying palate formation [11]. Studies in murine models have mapped the spatial and temporal dynamics of palatal cell populations, revealing distinct mesenchymal and epithelial domains associated with ossification, tooth development, and tissue remodeling [24]. Key signaling pathways, including Sonic hedgehog, BMP, fibroblast growth factor (FGF), transforming growth factor-β (TGF-β), and Wnt, coordinate the outgrowth, elevation, and fusion of palatal shelves, with perturbations leading to congenital anomalies such as cleft palate (CP) [11].

Embryology

The embryonic pharynx forms the framework around which the palate develops [2]. Neural crest cells aggregate around the pharynx, forming the pharyngeal arches from which the head and neck muscles, vasculature, and skeletal features will grow. The maxillofacial structures are derived from the first pharyngeal arch, which has both maxillary and mandibular neural crest cells. This provides a possible cause for the interdependence of maxillary development on the progression of mandibular development [25].

The maxillary position relative to the cranial base progresses in the later embryonic phases and depends on the chondrocranium and positioning of Meckel’s cartilage, which serves as the developing skeleton [26]. The developing MPS is subdivided into the frontal, middle, and dorsal sections. The former joins the incisive bones, the middle section joins the palatine processes, and the dorsal section connects the palatine bones [27]. The mandible develops at a faster rate than the nasomaxillary complex and descent of the palatal shelves; it has been noted that the discrepancy in the rate of ascent of palatal shelves is mediated by the chondrocranium and positioning of Meckel’s cartilage [25,26]. Development of the MPS can also be classified into two stages: the embryonic stage and the fetal stage. During the embryonic stage, the palatal shelves of the nasomaxillary complex meet on a horizontal plane, and the MPS begins to converge between the two bony shelves along a straight line [26,27]. The fetal stage is when interdigitation along the sutural line occurs [27]. A suture becomes detectable at 10 and a half weeks of gestation and is fully established at 12 weeks [28].

Development

Development of the suture is brought on by several mechanisms of growth-bone remodeling, sutural growth, and appositional growth. Bone remodeling is the simultaneous resorption of the ipsilateral surface and deposition/build-up on the contralateral surface [29]. Remodeling accounts for some of the changes observed; growth also occurs along sutures, which are more closely associated with the primary growth centers [27]. The primary growth centers in the palatal processes of the maxillae initiate ossification, and this process contributes to the axial growth of the suture [30].

Grossly, many factors operate in concert to coordinate palatal closure. The length of Meckel’s cartilages and the orientation of the maxillae to the cranial base influence the unfurling of the head and upper jaw relative to the primary curvature of the fetus [2]. Researchers hypothesize that a primitive cartilaginous skeleton precedes ossification and subsequent palatal development; cartilage cells have been found in early sutural structures [31]. It is believed that palatal fusion begins to occur at approximately 56 days of gestation [32].

Postnatally, the changes in the MPS can be classified into three stages with characteristic morphology. During the infantile stage, the MPS appears broad and “Y-shaped,” with the vomer between the maxillae [33]. The MPS has a wavy quality during the juvenile period [34]. During adolescence, there is increasing interdigitation along the sutural line, and distinct layers within the connective tissue emerge [33].

**Figure 2:**
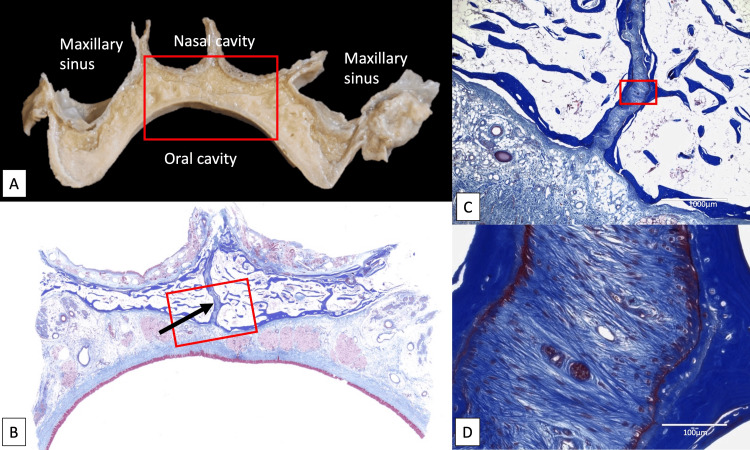
Coronal section of the maxilla at the first molar region. A: Gross anatomical observation B: Histological observation of the rectangular area of A (Masson’s Trichrome staining). The median palatine suture (arrow) is observed. C: Histological observation of the rectangular area of B (Masson’s Trichrome staining) D: Histological observation of the rectangular area of C (Masson’s Trichrome staining). Severe fibrotic change, regular fiber orientation, osteoblasts, and fibroblasts are observed in the suture. Source: Original images were obtained from cadaveric specimens in the present study.

The MPS continues to develop until 15 to 17 years of age, but perhaps is not solidified into one’s 30s [33]. Resorption occurs at the nasal surface of the hard palate until 14-15 years old, leaving resting lamellar bone, while appositional growth occurs at the oral surface of the palate until 13-14 years old. After 15/17 years (female/male), both the TPS and MPS were found to contain inactive osteoblasts. However, Persson et al. concluded that more factors than age strongly influence the start and the advancement of suture closure (Figure 2) [35].

Molecular and cellular biology of sutural dynamics

Recent investigations have identified critical molecular regulators of MPS development. Among them, Kysine methyltransferase 2D/mixed-lineage leukemia 4 (MLL4/KMT2D) has been shown to orchestrate both chondrogenic and osteogenic differentiation during palatal growth [36]. Single-cell RNA sequencing (scRNA-seq) has further revealed the existence of Piezo2-expressing chondrogenic mesenchymal cells, implying a mechanosensory role in maintaining suture patency [37]. The application of mechanical force activates Glioma-associated oncogene 1 (Gli1-positive) suture stem cells, wherein Wnt/β-catenin signaling is essential for the ensuing bone remodeling and expansion-induced osteogenesis [8-10]. Furthermore, extracellular vesicles derived from M1 macrophages have been shown to enhance bone turnover and inflammation during sutural expansion [38]. Other molecular effectors, such as the fat mass and obesity-associated protein (FTO) and microRNA-21, are also implicated in osteogenic differentiation and bone remodeling in response to mechanical stimuli [39,40]. These findings carry direct clinical implications, informing the optimal timing and methodology for maxillary expansion procedures. Nevertheless, the translation of these molecular insights into routine clinical practice remains constrained by a lack of standardized, validated imaging protocols and the moderate reliability of existing staging systems [41-44].

Imaging and assessment of the MPS

CT and CBCT are established as the gold standard for non-invasive assessment of MPS maturation, enabling classification into five distinct stages (A-E) based on morphological features [12-15]. Recent advancements in non-ionizing imaging, particularly high-frequency ultrasonography (HFUS) and 3-Tesla magnetic resonance imaging (3T MRI), have shown considerable promise for the anatomical assessment of musculoskeletal structures like the MPS. Ex vivo and pilot clinical studies have validated HFUS for accurately delineating MPS patency, demonstrating a strong correlation with micro-CT and CBCT findings [45-47]. This high spatial resolution, coupled with the absence of ionizing radiation, positions HFUS as a valuable tool for both initial diagnosis and longitudinal monitoring, especially in pediatric and adolescent populations where minimizing radiation exposure is critical [45,48,49]. Superior soft tissue contrast and multiplanar capabilities of 3T MRI enable detailed visualization of both hard and soft tissues without radiation risks, making it ideal for repeated evaluations during growth or treatment [49-52]. While MRI offers enhanced diagnostic utility through true volumetric imaging, practical limitations such as cost, scan duration, and accessibility persist [49,50]. Quantitative imaging metrics, such as suture density ratio and fractal analysis, offer objective measures of suture maturity and can predict the skeletal response to rapid maxillary expansion (RME) [53-55]. Moreover, artificial intelligence (AI)-based methods, particularly convolutional neural networks (CNNs), have achieved high accuracy in automating suture stage classification from CBCT images [56]. Building upon this foundation, next-generation models that integrate Vision Transformers (ViT) with CNNs have demonstrated even greater classification accuracy, reportedly surpassing that of experienced clinicians [57]. Furthermore, machine learning algorithms are increasingly leveraging image fusion techniques to synthesize information from multiple tomographic sections, enabling a more comprehensive and holistic evaluation of sutural maturation [58]. While these imaging advances have significantly improved the objectivity and accuracy of suture assessment, challenges persist in correlating imaging findings with true biological maturity and reliably predicting treatment outcomes [43,54-56]. The well-documented weak correlation between chronological age and suture maturation underscores the necessity for individualized imaging assessment, especially in adolescent and adult patients [43,59-61]. Furthermore, patient-specific anatomical factors such as palatal vault height warrant consideration. A lower vault is weakly negatively correlated with more advanced sutural fusion and may present anatomical constraints for certain interventions [62,63]. Although a direct link to ethnicity requires further investigation, this finding reinforces the imperative for individualized assessment. Although quantitative metrics and AI hold considerable promise for future standardization, rigorous validation against both histological and clinical outcomes is imperative [56,64].

Forensic age estimation from palatal suture obliteration

The progressive obliteration of the MPS has become a valuable tool for age estimation in both anthropological and medico-legal contexts [16-18]. This process occurs as calcified tissue accumulates within the planes of interdigitation, leading to sutural fusion [35]. Early research established that this obliteration follows a predictable sequence, typically beginning near the incisive fossa, followed by the posterior segment (posterior to the TPS), and finally the intermediate segment [65]. Studies have also noted that the palatine suture tends to obliterate more slowly than cranial vault sutures, offering a distinct timeline for skeletal assessment [16].

Building on this biological foundation, numerous large-scale studies employing postmortem CT and CBCT have quantitatively demonstrated a significant correlation between the extent of MPS closure and chronological age [18,66-68]. Regression models developed from these imaging modalities have achieved mean absolute errors typically ranging from 9 to 15 years [66,67]. However, the accuracy of these models is influenced by population and sex. For instance, research has highlighted regional and sex-specific variations in Chinese cohorts and confirmed the necessity of population-specific regression equations for Japanese and European groups, as the rate and pattern of suture obliteration can differ significantly [18,66-68].

Recent advances in machine learning have further enhanced the accuracy of age estimation from MPS maturation. By integrating MPS closure data with other skeletal indicators, these models can provide more precise age predictions, particularly in younger and middle-aged adults, without necessitating additional radiographic exposure [58]. Despite these advanced techniques, the reliability of using the MPS for age estimation diminishes in older adults. Increased inter-individual variability and the potential for incomplete fusion even at advanced ages mean that identifying age past 60 years becomes progressively less specific, though the length of the sutural opening can still provide insights [16,66,68,69].

Pathology

A median palatine cyst (MPC) can occur along the MPS and is of nonodontogenic origin. On imaging, the MPC is circular and located anteriorly in the palate, but may extend as far back as the molars. Due to the location of the cyst, the teeth may be displaced. Due to the variable location of the MPC, it can be confused with the nasopalatine duct cyst. These cysts may result from embryological issues, namely epithelial remnants along the MPS [70]. The pathogenesis of this condition is a subject of ongoing debate. It is commonly believed to originate from the entrapment of epithelial remnants within the MPS, which forms at the fusion site of the two lateral maxillary processes that constitute the hard palate [1,71]. A CP presents with a defect in the roof of the oral cavity and may expose the nasal cavity. Individuals with CP present with a narrow and/or irregular palatal vault; individuals typically have a higher anterior palate roof. This is due to a defect in the fusion of the palatine processes of the maxillae [72]. This may be attributed to maldevelopment of Meckel’s cartilage, a lack of sutural growth of the palatine processes, failure of the tongue to descend, or anomalous activity in the factors implicated in oronasal development [2]. A CP is generally surgically corrected.

Cleft lip and palate (CLP): a failure of the midline fusion cascade

CLPs are prevalent congenital anomalies resulting from failures in the intricate cascade of midline fusion during embryogenesis. This process is orchestrated by a tightly regulated interplay of genetic, epigenetic, and molecular factors, with disruptions leading to the spectrum of clefting phenotypes [73-77]. Key regulatory genes such as MLL4/KMT2D and colony-stimulating factor 1 receptor (CSF1R) are critical for midline development, and their disruption impairs chondrogenesis and widens the MPS, features evident in both syndromic and non-syndromic CLP [73,78,79].

The fusion of the palatal shelves is a multi-step process governed by a network of signaling pathways, including TGF-β, BMP, and WNT, which regulate the behavior of neural crest-derived cells [74,76,80,81]. Epigenetic regulation, particularly via non-coding RNAs, has emerged as a crucial control layer, modulating gene expression and mediating the effects of environmental teratogens [80-82]. The interplay between genetic susceptibility and environmental exposures is exemplified by gene-environment interaction models, such as the two-hit model involving Cadherin 1 (CDH1) variants and pro-inflammatory conditions [82].

scRNA-seq has revolutionized the field by identifying discrete cell populations at critical fusion sites and mapping their unique transcriptomic signatures during normal and aberrant development [83,84]. These high-resolution datasets have revealed novel candidate genes and regulatory networks involved in intramembranous ossification and mechanotransduction within the MPS [83,84].

Animal models, particularly genetically engineered mice, have been indispensable for dissecting these mechanisms and have recapitulated many aspects of human clefting by modeling mutations in key transcription factors and signaling components [76,84-87]. Coupled with genome-wide association studies (GWAS), this research has expanded the catalog of risk loci for CLP [73,79,88,89]. Despite these advances, significant gaps remain. A comprehensive understanding will require continued interdisciplinary research leveraging single-cell technologies, animal models, and human genetics to bridge the gap between molecular mechanisms and clinical outcomes [73,74,80].

Intervention: MARPE, RME, and surgically assisted rapid palatal expansion (SARPE)

In skeletally mature patients, the therapeutic approach to maxillary expansion has been reshaped by advanced imaging and minimally invasive techniques. CBCT assessment of MPS maturation is now a crucial diagnostic step, guiding the selection between MARPE, traditional RME, or SARPE [19,20,90]. These modalities are distinguished by their fundamental biomechanical principles and clinical indications. RME is a conventional, tooth-borne technique where expansion forces are applied to the teeth and transmitted to the suture [91,92]. It is primarily effective in growing patients with patent or minimally fused sutures, but it can produce undesirable dental side effects like buccal tipping in patients with increased sutural resistance. MARPE is a non-surgical, bone-borne approach that utilizes palatal microimplants to apply force directly to the maxillary bones [92,93]. By anchoring in bone, MARPE bypasses the dentition, thereby minimizing dental side effects and transmitting forces more effectively to the skeletal structures. This often results in a more parallel, bodily movement of the maxillary halves and has expanded the indications for non-surgical expansion into late adolescence and adulthood. SARPE is an invasive procedure reserved for skeletally mature patients with highly interdigitated or fused sutures [92,94]. It involves surgical osteotomies of the maxilla (e.g., Le Fort I osteotomy, midline palatal suture separation, and pterygomaxillary disjunction) to mechanically reduce skeletal resistance before the expansion appliance is activated. While highly effective, it is associated with greater morbidity, cost, and recovery time compared to MARPE. MARPE has proven particularly effective in late adolescents and adults, successfully splitting the MPS and often the pterygopalatine suture to achieve parallel skeletal expansion [95]. Predictors for success include a less advanced suture maturation stage, younger age, and shorter palatal length [19,96].

Comparative studies and systematic reviews consistently highlight MARPE's advantages over tooth-borne RME, which is less effective and prone to adverse dental effects in mature patients [19,20,96]. By utilizing bone-borne anchorage, MARPE transmits forces directly to the basal bone, maximizing skeletal effects while minimizing dental tipping and providing better vertical control - a key benefit in hyperdivergent patterns [20,97]. Consequently, MARPE can often obviate the need for SARPE, thereby reducing surgical morbidity, cost, and patient discomfort while still achieving clinically significant expansion and improvements in nasal airway function [96-99].

Nevertheless, the success of MARPE is not absolute. Advanced suture ossification may still necessitate SARPE, which remains the gold standard for highly resistant cases [20,96]. The importance of individualized appliance design and bicortical anchorage, optimized through digital workflows, is also well-documented [20]. While minor complications like local inflammation can occur, they are generally manageable [97]. In conclusion, the integration of CBCT-based diagnostics and MARPE has substantially expanded the therapeutic window for non-surgical maxillary expansion in skeletally mature individuals, offering a predictable and effective alternative to more invasive procedures [19,20,96,97].

Recommendations and future directions

Future investigations of the MPS should leverage high-resolution imaging modalities to elucidate its microstructural changes across different age groups and pathological states. Well-designed longitudinal cohort studies are needed to clarify the precise timeline of sutural maturation and obliteration, thereby refining age-estimation techniques for forensic applications. Furthermore, histological and molecular analyses of the MPS in pathological conditions, including CP and MPCs, may unveil the underlying mechanisms of aberrant fusion or remodeling. From a clinical standpoint, the integration of three-dimensional digital modeling into surgical planning could optimize interventions like RME and alveolar bone grafting. Finally, establishing standardized assessment protocols for MPS morphology is critical to facilitate robust cross-study comparisons and enhance the translational relevance of future research.

## Conclusions

The MPS represents a critical structural component of craniofacial anatomy, serving as both a growth site during development and a pivotal landmark for clinical interventions. Its morphological evolution from the embryonic period through adulthood provides not only valuable information for age estimation but also has direct implications for orthodontic and surgical procedures. A thorough understanding of its developmental trajectory, pathological variations, and biomechanical properties is therefore essential for optimizing patient outcomes across the disciplines of maxillofacial surgery, orthodontics, and forensic analysis.
